# Efficacy of 10% lidocaine gel for injection site pain associated with treprostinil in the treatment of pulmonary hypertension: a report of four cases

**DOI:** 10.1186/s40981-025-00834-4

**Published:** 2025-11-29

**Authors:** Shuji Kawamoto, Mariko Miyao, Kotaro Sakurai, Karin Kato, Makiko Ikeura, Akiko Hirotsu, Hideyuki Kinoshita, Moritoki Egi

**Affiliations:** 1 Department of Anesthesia, Kyoto University Hospital, 54 Shogoin Kawahara-cho, Sakyo-ku, Kyoto, 606-8507 Japan; 2 Patient safety unit, Kyoto University Hospital, Kyoto, Japan; 3 Department of Community Medicine Supporting System, Kyoto University Graduate School of Medicine, Kyoto, Japan

**Keywords:** Pulmonary arterial hypertension, Treprostinil, Injection site pain, Lidocaine gel

## Abstract

**Background:**

Treprostinil, a prostacyclin analogue, is an effective treatment for pulmonary arterial hypertension (PAH). However, continuous subcutaneous infusion is often complicated by severe injection site pain, which can limit continuation of therapy.

**Case presentation:**

We report four PAH patients who developed severe injection site pain during subcutaneous treprostinil therapy in the lower abdomen. A hospital-compounded 10% lidocaine gel was applied around the infusion site. Pain was markedly reduced in all cases, with visual analog scale scores improving from 75 to 100/100 to 0–20/100. In two patients, tramadol could be reduced or discontinued, and all patients were able to continue treprostinil therapy without pain-related limitation or treatment interruption. No systemic adverse effects were observed, although one patient experienced mild local skin irritation.

**Conclusions:**

Topical 10% lidocaine gel provided rapid and effective relief of injection site pain associated with subcutaneous treprostinil, facilitating continuation of therapy without pain-related interruption in patients with PAH.

**Supplementary Information:**

The online version contains supplementary material available at 10.1186/s40981-025-00834-4.

## Background

Pulmonary arterial hypertension (PAH) is a progressive disease in which endothelial dysfunction leads to reduced production of prostaglandin (PG) I₂, a vasodilator, resulting in predominance of vasoconstriction and platelet aggregation, and subsequently increased pulmonary vascular resistance [1]. Treprostinil, a PGI₂ analogue, exerts vasodilatory and antiplatelet effects and has been shown to improve exercise tolerance and prognosis in patients with PAH [2, 3]. In addition to intravenous administration, subcutaneous infusion is available and suitable for home therapy. However, injection site pain occurs at a very high frequency, often necessitating treatment discontinuation or a switch to another route of administration [4–7]. Although injection-site pain is a common adverse event during subcutaneous treprostinil therapy, previous reports have indicated that its severity does not necessarily correlate with the infusion rate [6, 8]. Treprostinil is generally administered as a continuous subcutaneous infusion for long-term management, often continuing for years unless discontinued due to adverse effects or disease progression. Conventional pain management strategies, including NSAIDs and opioids, have been employed, but these often provide insufficient analgesia and may be associated with systemic adverse effects. Lidocaine, a local anesthetic, inhibits voltage-gated sodium channels to block nociceptive transmission in peripheral nerves. Topical preparations have minimal systemic absorption and are therefore considered advantageous in terms of safety [9]. However, there have been few reports on the efficacy of high-concentration lidocaine gel for injection site pain associated with treprostinil. In this report, we describe four cases in which the application of 10% lidocaine gel to the site of subcutaneous treprostinil infusion resulted in remarkable pain relief, allowing patients to continue therapy without pain-related interruption.

## Case presentation

**Fig. 1:**
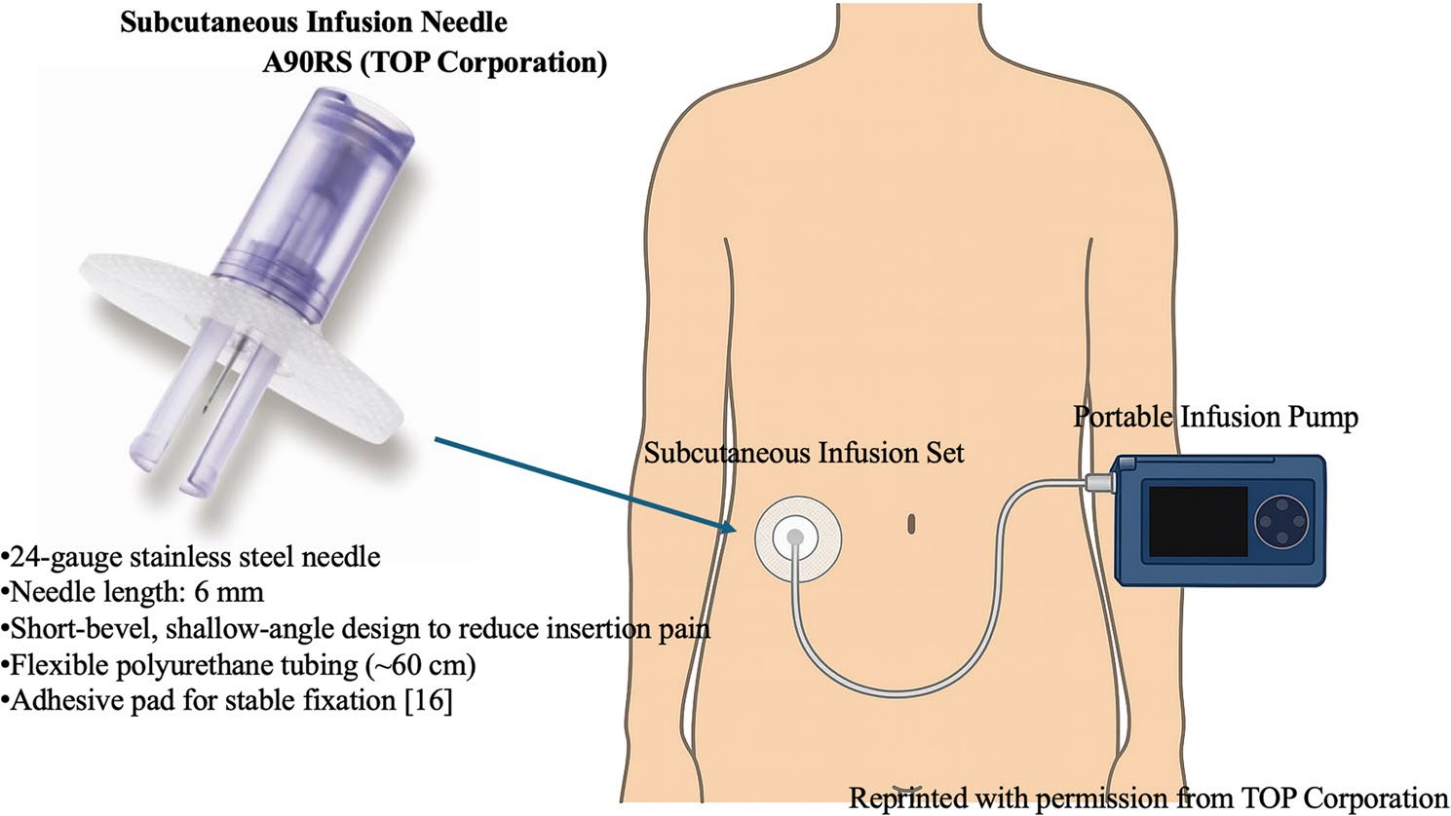
Subcutaneous infusion needle and infusion system used for treprostinil administration The figure shows the dedicated subcutaneous infusion needle (A90RS; TOP Corporation) and the infusion setup used for continuous subcutaneous treprostinil administration. A schematic illustration demonstrates the placement of the infusion needle in the abdominal subcutaneous tissue and its connection to the portable infusion pump. Formal permission to reproduce the photograph was obtained from TOP Corporation [16]

This work was approved by the institutional ethics committee (approval number R3381), and written informed consent for both study participation and the use of the hospital-compounded 10% lidocaine gel was obtained from all patients. The subjects were four patients with PAH who were referred from the department of the circulatory oragans at our hospital to our department due to severe injection site pain associated with subcutaneous treprostinil infusion. All patients received treprostinil therapy and lidocaine gel treatment on an outpatient basis. Continuous subcutaneous treprostinil infusion was performed using a syringe infusion pump (TOP-8200RS; TOP Corporation, Tokyo, Japan) connected to a dedicated subcutaneous infusion needle (A90RS; TOP Corporation; 24-gauge, 6 mm length) (Fig. 1). In accordance with the manufacturer’s package insert, the infusion needle was inserted into the abdominal subcutaneous tissue [5]. Pain assessment was primarily performed using the visual analog scale (VAS), and the use of concomitant analgesics, feasibility of continuing treatment, and the occurrence of adverse events were evaluated. Patients visited the pain clinic once a month, and the average pain intensity during the preceding month was evaluated using VAS. Approximately 1–2 g of the 10% lidocaine gel was applied around the infusion site twice daily, and the gel was wiped off 30–60 min after each application. The total amount used was limited to one 50 g jar per month as a guideline. Analgesia was typically achieved within 30–60 min after application, and the analgesic effect was sustained for approximately 4–8 h in most cases, and additional applications beyond the twice-daily schedule were not permitted. A summary of the clinical courses of the four cases is shown in Table 1. The 10% lidocaine gel used in this report was compounded in our hospital pharmacy as a special preparation that is not commercially available. Details of the gel preparation and application procedures are described in the Supplementary Material.

**Table 1:** Clinical summary of four cases

Case	Age/Sex	Underlying disease	VAS before LG	VAS after LG	Analgesic change	Adverse events	Treprostinil dose (Before→After)
1	30s/F	SLE-associated PAH	75	10	Tramadol reduced 8→2 tablets/day	None	2.5 µg/h →7.5 µg/h Improvement in heart failure symptoms
2	20s/F	Idiopathic PAH	80	20	No reduction of analgesics	None	16.5 µg/h → discontinued due to disease progression, switched back to IV epoprostenol
3	40s/F	MCTD with severe PAH	100	20	No reduction of analgesics	None	16.5 µg/h → maintained
4	50s/F	Idiopathic PAH	100	20	Tramadol discontinued	Mild local skin irritation	24 µg/h → maintained

*SLE* Systemic lupus erythematosus, *MCTD* Mixed connective tissue disease, IV Intravenous, *LG* Lidocaine gel, *PAH* Pulmonary arterial hypertension, *HOT* Home oxygen therapy, *VAS* Visual analog scale

### Case 1

A woman in her 30 s with severe PAH secondary to systemic lupus erythematosus (SLE) was followed by the cardiology and rheumatology departments. After the initiation of continuous subcutaneous treprostinil infusion, she reported persistent severe pain at the infusion site, with a VAS score of 75/100. The pain interfered with daily activities such as walking and dressing, and her Pain Disability Assessment Scale (PDAS) was 23/60. She was taking up to eight tablets per day of a tramadol/acetaminophen combination (each tablet containing tramadol hydrochloride 37.5 mg and acetaminophen 325 mg), but pain control was inadequate, and the severe discomfort limited the clinical feasibility of further dose escalation. After referral to our department, 10% lidocaine gel was applied to the infusion site, leading to a reduction of the VAS score to 10 within a few days and improvement of the PDAS score. The tramadol/acetaminophen dosage was reduced to two tablets per day, and treprostinil dose escalation improved her heart failure symptoms. No adverse events were observed.

### Case 2

A woman in her 20 s with idiopathic PAH had previously been treated with intravenous epoprostenol via a central venous catheter. Following catheter-related infection, her therapy was switched to subcutaneous treprostinil. After the switch, she experienced widespread abdominal pain at the infusion site, with a VAS score of 80/100 and a PDAS score of 43/60. The pain caused significant difficulty in maintaining employment, and the patient strongly requested discontinuation of therapy. NSAIDs and four tablets per day of tramadol/acetaminophen were ineffective. After introduction of 10% lidocaine gel at our department, the VAS score improved from 80 to 20, and no adverse effects occurred. However, her underlying disease subsequently worsened, and a Hickman central venous catheter was inserted to switch therapy back to intravenous epoprostenol.

### Case 3

A woman in her 40 s developed mixed connective tissue disease (MCTD) at age 23 and subsequently severe PAH at age 32. She was on home oxygen therapy (2 L/min). After initiation of subcutaneous treprostinil, she experienced severe persistent pain at the infusion site, with a VAS score of 100/100. The pain was pronounced even at rest, leading to sleep disturbance and depressive symptoms. She was taking tramadol/acetaminophen (four tablets per day), diclofenac capsules, and 600 mg of acetaminophen, but pain control remained insufficient. After starting 10% lidocaine gel, her VAS score improved to 20, and nocturnal pain was relieved, leading to improved sleep quality. The patient reported that nocturnal awakenings due to pain were no longer observed. Although existing analgesics could not be reduced, no adverse effects were observed, and gradual tapering was considered feasible. Despite being under evaluation for lung transplantation, continuation of treprostinil therapy was possible.

### Case 4

A woman in her 50 s with idiopathic PAH had been receiving continuous subcutaneous treprostinil infusion. However, she experienced severe pain at the time of needle replacement, and the pain persisted at a VAS score of 100/100 for approximately 10 days after each replacement. This markedly restricted her daily activities, and she required tramadol/acetaminophen combination tablets (four tablets per day). At her initial visit to our department, her VAS score was 100/100 and her PDAS score was 30/60. After the introduction of lidocaine gel, the VAS decreased to 20, and the period of tramadol/acetaminophen use after needle replacement was shortened to 7 days. Ultimately, oral tramadol/acetaminophen was discontinued. During the course of treatment, she developed mild skin irritation at the injection site, but no serious adverse events occurred.

## Discussion

Subcutaneous administration of treprostinil can be managed at home and avoids the risks associated with central venous catheter infections, making it a valuable therapeutic option [8, 10]. However, injection site pain is an extremely frequent adverse event and represents a major obstacle to long-term treatment continuation. In a Japanese clinical trial, all patients experienced injection site pain or local reactions such as erythema and swelling, with approximately 42% requiring a switch to intravenous administration and about 13% discontinuing treatment altogether [4]. Similarly, Barst et al. reported that 92% of patients with long-term subcutaneous treprostinil experienced injection site pain, and 23% of them needed to discontinue therapy due to pain [3], underscoring that pain management is one of significant clinically issues.

Although injection-site pain is common during subcutaneous treprostinil therapy, previous studies, including those by White et al. [6] and Simonneau et al. [8], have shown no clear correlation between infusion rate and pain severity. In our four cases, severe pain occurred irrespective of treprostinil dose, supporting the notion that pain intensity is not dose-dependent but may be influenced by individual or local factors. While pain itself is not determined by dose, uncontrolled local pain can still limit the clinical feasibility of treatment continuation.

Although the mechanisms underlying this pain are multifactorial, treprostinil, as a PGI_2_ analogue, is thought to activate prostacyclin receptors on nociceptors and lower the pain threshold, accompanied by local inflammatory responses [2]. Therefore, a locally acting sodium channel blocker capable of suppressing both nociceptive transmission and inflammation is theoretically suitable for this pain. Existing pain management strategies, such as NSAIDs or opioids, often fail to provide sufficient analgesia and may produce systemic adverse effects. Hence, a safe topical approach that blocks nociceptive transmission directly at the infusion site is desirable.

We selected a 10% lidocaine gel because lower concentrations (2–5%) generally provide only limited surface anesthesia, as their lower lipid solubility restricts diffusion through tissue barriers and results in a shorter duration of action [9]. Recent studies have indicated that 10% lidocaine formulations, including spray and gel, provide faster and more effective analgesia than lower-concentration preparations, with no evidence of increased systemic absorption [11, 12]. These findings suggest that a higher concentration may enhance transcutaneous diffusion and allow analgesic effects to reach nociceptive terminals in the dermis and subcutaneous tissue where treprostinil infusion provokes pain, while systemic absorption from localized, time-limited application to intact skin is expected to remain within a safe range, and thus well below concentrations associated with toxicity. Therefore, the use of a 10% formulation was considered pharmacologically reasonable for this setting.

The analgesic mechanism of lidocaine involves blockade of voltage-gated sodium channels, reducing ectopic firing and abnormal spontaneous activity [13, 14]. Additionally, the hydrophilic base of the compounded gel may also enhance dermal diffusion and local retention, supporting continuous analgesic efficacy during repeated application. These multimodal local actions likely explain the rapid and sustained pain relief observed in our patients.

In the four cases presented, topical 10% lidocaine gel markedly reduced VAS scores, allowed tapering or discontinuation of existing analgesics. Importantly, in all cases, treatment discontinuation due to pain was avoided. In one patient, VAS improved to zero, demonstrating an effect that appears to exceed that of previously reported adjunctive measures. One patient developed mild local skin irritation, but overall tolerability was favorable.

Clinically, these outcomes indicate that mechanism-based local analgesia can restore treatment adherence and daily functioning in PAH patients receiving subcutaneous treprostinil. Because treprostinil dose correlates with long-term survival [15], effective pain management may indirectly contribute to improved prognosis.

Although this report is limited by the small number of cases, it provides initial clinical evidence supporting the use of high-concentration lidocaine gel for treprostinil-induced pain. Further clinical investigations, including prospective trials, will be necessary to confirm its efficacy and safety.

In conclusion, we report four cases in which high-concentration lidocaine gel was associated with marked improvement of injection site pain during subcutaneous treprostinil therapy. These observations suggest that topical lidocaine gel may be a useful option for pain management in　patients with treprostinil-related injection site pain.

## Supplementary Information

Supplementary Material 1

Supplementary Material 2

## Data Availability

All data generated or analyzed in the study are included in this article.
