# Supplementary material for: Efficacy of 10% lidocaine gel for injection site pain associated with treprostinil in the treatment of pulmonary hypertension: a report of four cases
Source: JA Clin Rep. 2025 Nov 29;12:2. doi: 10.1186/s40981-025-00834-4 (PMC12770123; doi:10.1186/s40981-025-00834-4)
Supplement: Supplementary file 1 — Supplementary Material 1 [file 40981_2025_834_MOESM1_ESM.docx]

**Preparation, Storage, and Usage of 10% Lidocaine Gel**

The 10% lidocaine gel used in this report was prepared as a hospital-compounded formulation based on previously published methods.

**Preparation**

- 5.5 g of diisopropanolamine (Nacalai Tesque, Kyoto, Japan) and 5 g of HIVISWAKO 104 (Wako Pure Chemical Industries, Osaka, Japan) were dissolved in 100 mL of purified water, allowed to swell, and then mixed with an additional 150 mL of purified water until homogeneous.
- To this solution, 48 mL of propylene glycol (Maruishi Pharmaceutical, Osaka, Japan), 50 g of lidocaine (Wako Pure Chemical Industries, Osaka, Japan) dissolved by gentle heating, and 150 g of ethanol (Maruishi Pharmaceutical, Osaka, Japan) were gradually added.
- The mixture was stirred thoroughly until uniform gelation was achieved.
- The final 10% lidocaine gel was dispensed into 50 g sterile ointment jars (MI Chemical, Japan).

**Storage**

- The product was stored at room temperature.
- Shelf-life was set at one year.

**Usage**

- Patients were instructed to apply approximately 1–2 g of the 10% lidocaine gel to the infusion site twice daily.
- The gel was wiped off 30–60 minutes after each application.
- The total amount used was kept within one 50 g jar per month as a guideline.
