# Supplementary material for: Efficacy of 10% lidocaine gel for injection site pain associated with treprostinil in the treatment of pulmonary hypertension: a report of four cases
Source: JA Clin Rep. 2025 Nov 29;12:2. doi: 10.1186/s40981-025-00834-4 (PMC12770123; doi:10.1186/s40981-025-00834-4)
Supplement: Supplementary file 2 — Supplementary Material 2 [file 40981_2025_834_MOESM2_ESM.pdf]

Shuji Kawamoto <skawamot@kuhp.kyoto-u.ac.jp>

（株式会社トップ_中原と申します）弊社ホームページへのお問合せについて

株式会社トップ　中原秀敏 <nakahara@top-tokyo.co.jp> 2025年10月30日 14:16
To: Shuji Kawamoto <skawamot@kuhp.kyoto-u.ac.jp>

京都大学医学部附属病院
麻酔科　川本修司 先生御机下

お忙しいところ、貴重な論文をお送り頂きまして誠にありがとうございます。
関連部署に急ぎ確認のうえ、ご使用内容に問題が無いことを確認致しました。
弊社製品の写真を是非ともご使用くださいませ。

患者様の注射部位の痛みには、弊社留置針とチューブの接続の際に生じる刺激が関連する部分もございますので、弊社と致しましては少しでも患者様がご使用しやすい、より良い製品のご提供ができるよう努めて参ります。

==

株式会社トップ
営業本部　営業推進室
中原 秀敏

〒120-0035
東京都足立区千住中居町19-10
TEL：03-3882-7741
FAX：03-3882-7744
E-mail：nakahara@top-tokyo.co.jp
携帯電話：080-1366-7675

----- Original Message -----

> トップ株式会社　中原様
>
> この度は早々にご返信いただきありがとうございます。
> 京大麻酔科の川本です。
>
> 論文原稿と御社製品画像を転載させていただいた図を添付いたします。現在論文はJA Clinical reportsに投稿し査読を受けているところです。
> ご確認のほど何卒よろしくお願いいたします。
>
> 川本 修司 (Shuji Kawamoto)

京都大学医学部附属病院 麻酔科
〒606-8507　京都府京都市左京区聖護院川原町54
TEL:075-751-3433　FAX:075-752-3259
E-mail: s kawamot@kuhp.kyoto-u.ac.jp
sk.anpanman@gmail.com

2025年10月30日(木) 11:53 株式会社トップ　中原秀敏 <nakahara@top-tokyo.co.jp>:

> 京都大学医学部附属病院
> 麻酔科　川本修司 先生御机下
>
> いつも大変お世話になっております。
> 株式会社トップ 営業本部に所属しております中原と申します。
> この度は弊社ホームページにお問合せをいただき、誠にありが
> とうございます。
>
> 弊社が取り扱っております「携帯ポンプ用留置針A90RS」の写真を
> 先生がご投稿される学術誌へ資料としてご掲載したいとの内容に
> つきまして、社内規定による処理上、先生が仰られる掲載予定図
> のサンプルを頂戴できれば大変有難く存じます。
>
> お手数をお掛けして大変恐縮ではございますが、何卒、よろしく
> お願い申し上げます。
>
> ===

> 株式会社トップ
> 営業本部　営業推進室
> 中原 秀敏
>
> 〒120-0035
> 東京都足立区千住中居町19-10
> TEL：03-3882-7741
> FAX：03-3882-7744
> E-mail：nakahara@top-tokyo.co.jp
> 携帯電話：080-1366-7675
